# Predicting *ROS1* and *ALK* fusions in NSCLC from H&E slides with a two-step vision transformer approach

**DOI:** 10.1038/s41698-025-01037-x

**Published:** 2025-07-30

**Authors:** Eghbal Amidi, Mohammadreza Ramzanpour, Ming Chen, Tommy Boucher, Mukund Varma, Timothy Samec, Brian Lamon, Nicolas Stransky, Mark R. Miglarese, Matthew Oberley, David Spetzler, George W. Sledge

**Affiliations:** Caris Life Sciences, Phoenix, AZ USA

**Keywords:** Computational biology and bioinformatics, Molecular biology, Biomarkers, Molecular medicine, Oncology

## Abstract

Non-small cell lung cancer (NSCLC) is one of the deadliest and most prevalent cancers worldwide, with 5-year survival rates of ~28%. The molecular heterogeneity within NSCLC encompasses several types of genetic alterations, such as mutations, amplifications, and rearrangements, and can drive aggressive tumor behavior and poor response to therapy. Among these genetic alterations are *ALK* and *ROS1* fusions. Though these fusion events are relatively rare, their identification is crucial for selecting effective targeted treatments and avoiding therapies with significant side-effects. Fluorescent in situ hybridization (FISH), immunohistochemistry (IHC), and sequencing of DNA and RNA are standard methods to detect *ALK* and *ROS1* fusions, but they are costly, time-consuming, and require adequate tumor tissue. Here we employ deep learning models using whole slide images (WSIs) of hematoxylin and eosin (H&E)-stained formalin-fixed paraffin embedded (FFPE) NSCLC tumor specimens to identify tumors most likely to harbor *ALK* and *ROS1* fusions in a cohort of 33,014 patients, out of which 306 and 697 patients are positive for *ROS1* or *ALK* fusions, respectively. A vision transformer model (MoCo-V3) was trained as a feature extractor, followed by training transformer-based models to predict the presence of *ROS1* and *ALK* fusions. Due to the limited positive sample size for *ROS1*, a two-step specialized training procedure was implemented to enhance prediction performance during cross-validation. Our approach achieved receiver-operating characteristic areas under the curves (ROC AUCs) of 0.85 for *ROS1* and 0.84 for *ALK* on a holdout dataset, demonstrating the effectiveness of this method. This framework holds significant potential for clinical application by offering a scalable, accurate, and cost-efficient method for detecting *ALK* and *ROS1* fusions. Furthermore, it may serve as a pre-screening tool to identify candidates for confirmatory diagnostic testing and clinical trials, ultimately improving the efficiency of selecting appropriately targeted therapies for NSCLC patients.

## Introduction

In the United States, lung cancer ranks as the second most common type of cancer and the leading cause of cancer-related deaths^1,2^. It is projected that in 2025, there will be 226,650 new cases of lung cancer and 124,730 deaths attributable to this disease^2^. Lung cancer is broadly classified into two main histological subtypes: small cell lung cancer (SCLC) and non-small cell lung cancer (NSCLC). This study focuses on NSCLC, which constitutes ~80–85% of all lung cancers^2–4^.

Targeted therapy has become a cornerstone of cancer treatment, particularly for managing diseases like NSCLC, where the precise identification of genetic alterations can guide therapeutic strategies. A key aspect of this genetic evaluation involves the detection of mutations or rearrangements, such as fusions, of specific genes that drive cancer progression. Among these, gene fusions involving the anaplastic lymphoma kinase (*ALK*) and ROS proto-oncogene 1 (*ROS1*), though rare, are highly significant in the context of lung cancer diagnosis and treatment^5,6^. *ALK* and *ROS1* are involved in chromosomal rearrangements that result in the production of constitutively active, tumor-driving receptor tyrosine kinases, and patients with *ALK* or *ROS1* fusions are recommended specific targeted therapies. Identifying these genetic alterations is therefore pivotal for selecting the most effective treatment, underscoring the importance of precise genetic screening in clinical oncology^7–12^. The proposed model could significantly aid in early patient stratification for clinical trials by pre-screening likely *ALK* and *ROS1* fusion-positive cases, ensuring confirmatory testing resources are focused effectively.

Currently, there are several companion diagnostic tests for detecting *ROS1* and *ALK* fusions including fluorescent in situ hybridization (FISH) and immunohistochemistry (IHC). Next Generation Sequencing (NGS) is another potential way to identify *ROS1* and *ALK* fusions^13–16^. FISH and IHC are fast and relatively inexpensive, although they consume tissue which is not an efficient way of screening patient specimens for rare biomarkers. NGS allows for testing of a wider array of biomarkers but is costly and time consuming. Deep learning models developed on digital pathology images have surged in popularity within histopathology for image classification and predicting genetic mutations and rearrangements^17–26^. Most of these models are trained on widely utilized and economical hematoxylin and eosin (H&E) stained slides, presenting a potentially accessible method for biomarker detection where the costs of image acquisition are relatively inexpensive once the capital expense of scanners have been paid. Although H&E slides are widely available, each digital image is large, and annotating ground truth data for each cell or region is very labor-intensive and time-consuming for pathologists. In biomarker prediction, typically only slide-level labels are available, indicating whether a patient tests positive for a specific biomarker. To address this, multiple instance learning (MIL) models have been developed to train on these slide-level labels rather than detailed tile-level annotations^27–31^. These models incorporate an attention mechanism that helps identify and learn from the most significant regions of the slides, those that contribute most to the final predictions.

Recently, vision transformer-based models have gained prominence as effective attention-based tools in histopathology^32–35^. These models demonstrate considerable promise in detecting various biomarkers across multiple types of cancer. Vision transformer-based models typically include separate modules for feature extraction and feature aggregation. The feature extraction module is trained in a self-supervised manner without labels. Once trained, it can convert whole slide images (WSIs) into feature matrices. These matrices are then used to train the feature aggregation module, utilizing only slide-level labels.

Although deep learning models show significant potential for predicting various biomarkers, there are very few studies on their use for detecting *ROS1* and *ALK* rearrangements in NSCLC using H&E slides. This scarcity may be due to the low prevalence of these mutations—*ROS1* fusions occur in only 1–2% of NSCLC patients and *ALK* fusions occur in less than 5%^36^. A study by Mayer et al.^36^ reported encouraging Positive Percent Agreement (PPA) and Negative Percent Agreement (NPA) for these biomarkers but was constrained by its small sample size (234 cases, with 15 *ALK*-positive and 7 *ROS1*-positive). Terada^37^ also investigated *ALK* rearrangements but their study was similarly restricted by a small cohort size (66 *ALK*-positive and 142 negative cases) and a modest receiver operating characteristic area under the curve (ROC AUC) of 0.73. Coudray et al. focused on variants of 10 genes in lung adenocarcinomas, excluding *ROS1*, and the performance of their models did not translate to predicting *ROS* variants^38^. Tan et al.^39^ conducted a more extensive study with a larger dataset (54 positive and 834 negative *ALK* fusion positive cases for training, 66 positive and 1,398 negative for testing) achieving an impressive 0.92 in their test set; however, their models were trained using demographic data, pathology reports, and serum tumor markers, not by the much more facile approach described herein, using only H&E stained slides.

In this study, we analyzed a large NSCLC cohort of 33,014 patients, including 306 *ROS1*-fusion positive and 697 *ALK*-fusion positive cases (Table 1). We developed a deep learning pipeline based on vision transformer models trained on H&E-stained slides to predict the presence of *ROS1* and *ALK* fusions. To address the challenge of limited *ROS1*-positive cases, we adopted a two-stage training strategy: first training the model to detect a composite biomarker (*ROS1*, *ALK*, and *NTRK* fusions), then fine-tuning it specifically for *ROS1* or *ALK* prediction. This approach significantly enhanced performance in identifying *ROS1* fusions, particularly in an independent holdout cohort not used in training.

**Table 1:** Summary of positive and negative case counts for ROS1, ALK, NTRK, and RAN fusion biomarkers, presented across the total dataset (CV + Holdout), the cross-validation (CV) set, and the holdout set

	Total		CV		Holdout	
	positive	negative	pos	neg	pos	neg
ROS1	306	32,708	260	27,792	46	4916
ALK	697	32,317	589	27,463	108	4854
NTRK	25	32,989	23	28,029	2	4960
RAN	1,028	31,986	872	27,180	156	4806

The RAN label denotes samples positive for any ROS1, ALK, or NTRK fusion. The total number of unique samples across all sets is 33,014.

## Results

### Comparison of direct vs. two-stage training strategies

We compared the ROC AUC in the validation set for two training scenarios: (1) direct training for the target biomarker prediction, and (2) initial training on *RAN* followed by fine-tuning for the target biomarker. These scenarios are referred to as direct and train-finetune models, respectively. The *RAN* model, trained to predict the composite *RAN* label, achieved a maximum ROC AUC of 0.86 in the validation set. Building on this performance, we evaluated whether fine-tuning the *RAN*-trained model on individual biomarkers (the train-finetune approach) would improve predictive accuracy compared to direct training.

**Fig. 1:**
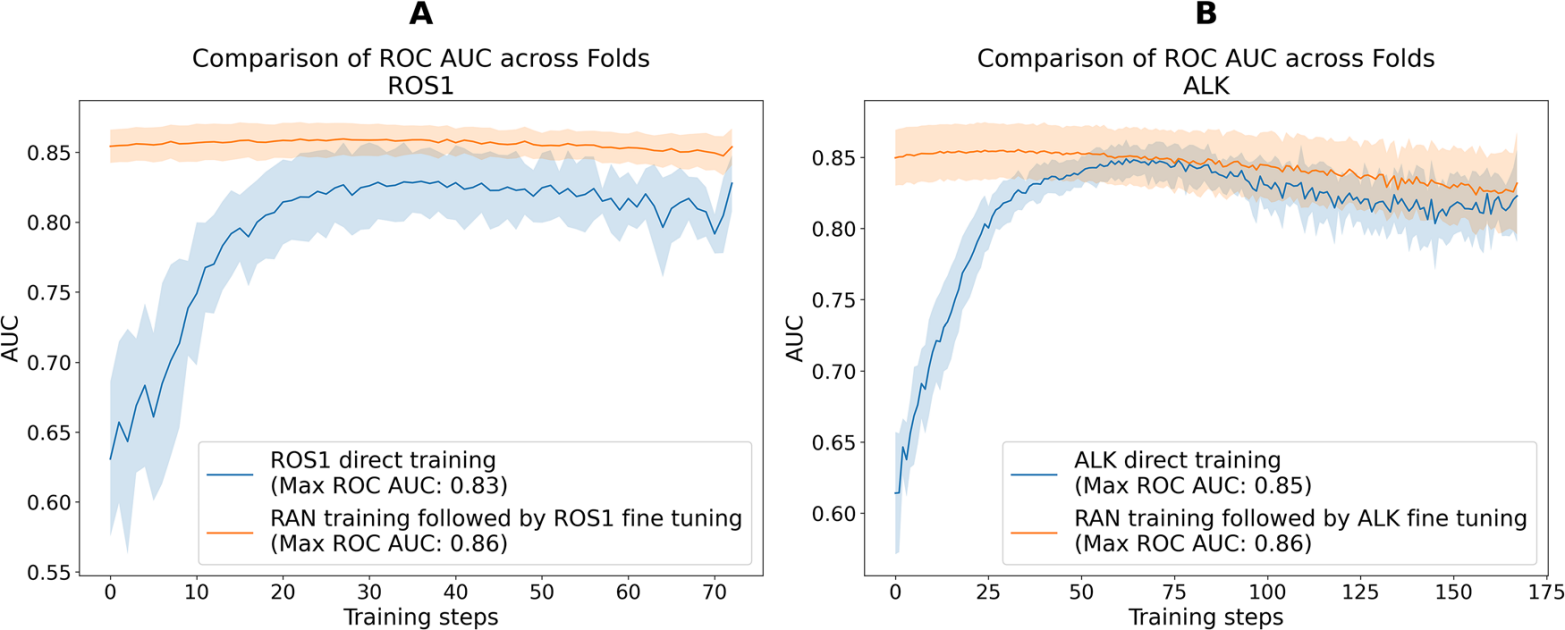
Performance comparison of direct versus the train-finetune approach for predicting specific biomarkers. The figure displays the validation Receiver Operating Characteristic (ROC) Area Under the Curve (AUC) values across cross-validation folds for two distinct training strategies. Blue lines represent models directly trained to predict the target biomarker (*ROS1* in (**A**) and *ALK* in (**B**)). Orange lines represent models initially trained on the RAN dataset and subsequently fine-tuned to predict the respective target biomarker. Shaded regions around the lines indicate the standard deviation across folds, providing a measure of variability in performance.

As illustrated in Fig. 1A, for the *ROS1* fusion biomarker, the ROC AUC for the train-finetune model is consistently superior to that of the direct model, achieving a ROC AUC of 0.86 compared to 0.83. In the train-finetune model, the ROC AUC increases during the early training steps and then exhibits a slight decline as training progresses. However, due to greater variability in the direct model’s ROC curve, the changes in the train-finetune model are less apparent. This smaller variation in the train-finetune model is expected, as the learning rate used for fine-tuning from *RAN* to *ROS1* fusions is ten times smaller than that used in the direct training scenario.

For *ALK* fusion*s*, shown in Fig. 1B, although the train-finetune model also produces a higher ROC AUC, the difference between the two models is not as substantial (0.86 vs. 0.85). Both models demonstrate stable performance trends throughout training, with less variability compared to *ROS1*.

### *ROS1* fusion model performance

**Fig. 2:**
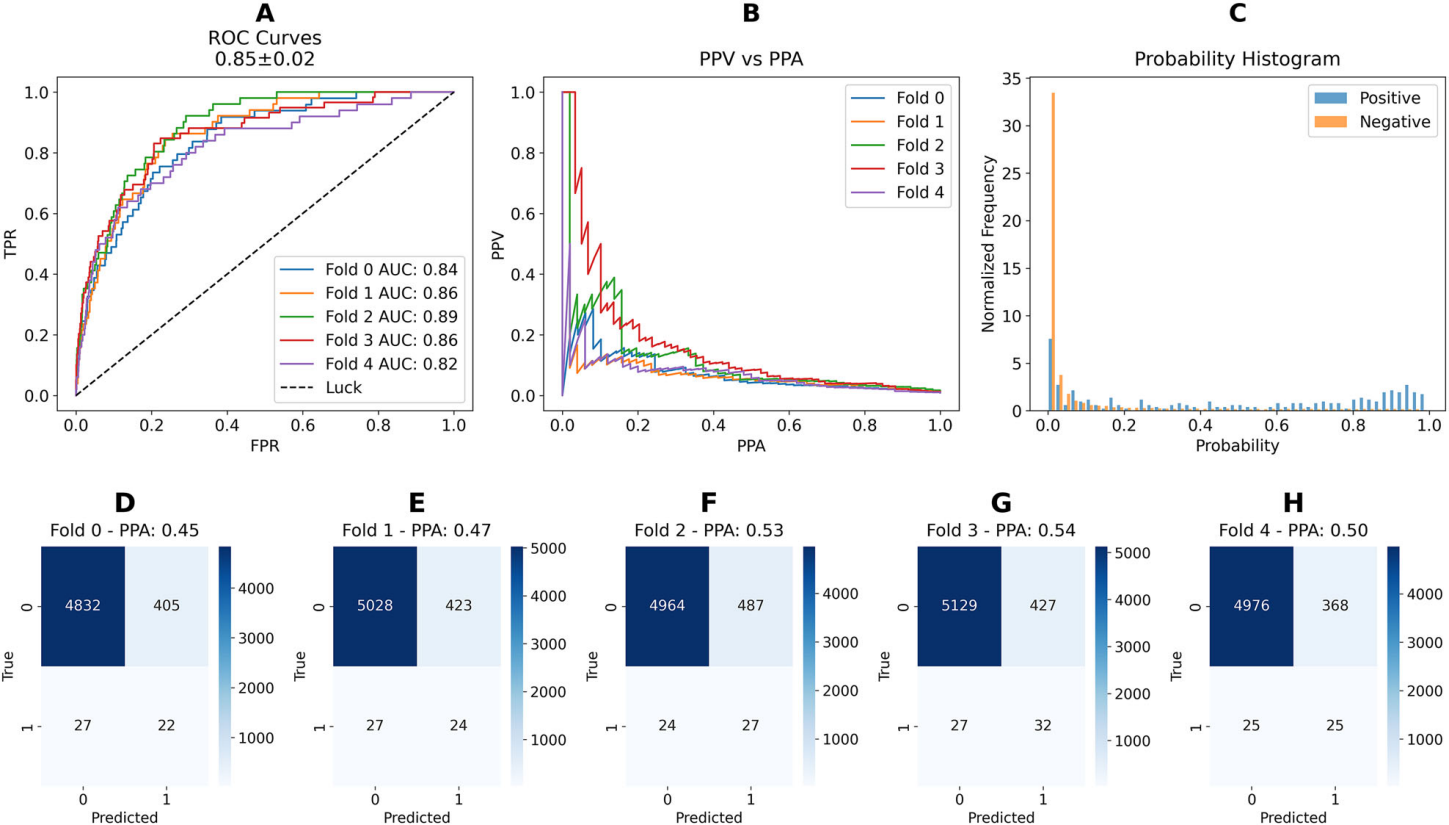
Performance plots for *ROS1* prediction on the test sets using the train-finetune model. **A** Receiver Operating Characteristic (ROC) Curves: This panel displays the ROC curves for each cross-validation fold, with individual ROC AUC values listed in the legend. The average ROC AUC (0.85) ± standard deviation (0.02) across all folds is shown at the top of the plot. **B** Precision-Recall Curves: This panel presents the precision-recall curves for each fold. Precision corresponds to Positive Predictive Value (PPV), and recall corresponds to Positive Percent Agreement (PPA). **C** Normalized Probability Histogram: This panel shows the normalized histogram of predicted probabilities for the test set, distinguishing between positive and negative predictions. **D**–**H** Confusion Matrices for Individual Folds: These panels display the confusion matrices for different cross-validation folds (Fold 0 through Fold 4, respectively). In each matrix, true labels are compared to predicted labels. The Positive Percent Agreement (PPA) is indicated at the top of each confusion matrix.

The performance plots for *ROS1* fusion prediction on the test datasets using the train-finetune model are shown in Fig. 2. The ROC curve in Fig. 2A, demonstrates an average ROC AUC value of 0.85 which, according to ref. ^40^, indicates excellent diagnostic significance, further supporting the clinical relevance of our model’s performance. However, due to the highly imbalanced dataset, the precision-recall curve is less compelling (Fig. 2B), with a mean area under the precision-recall curve (PR AUC) of 0.1 ± 0.03 across the five folds. While this curve is standard in machine learning for evaluating performance on imbalanced datasets, it is important to note that precision corresponds to Positive Predictive Value (PPV) and recall corresponds to Positive Percent Agreement (PPA) in diagnostic terms. The normalized histogram of the predicted probability values for *ROS1* fusion positivity is displayed in Fig. 2C, revealing a significant number of *ROS1* fusion positive cases (vertical blue bars) clustered near the left side of the histogram. This indicates a high prevalence of extreme false negative cases, which can be mitigated by assigning higher weights to positive cases in the loss function, although this adjustment may lead to an increase in false positives. The confusion matrices for the five different folds are shown in Fig. 2D–H, with the true positive rate (TPR) ranging from 0.45 to 0.54 for our model.

To further evaluate model robustness, we assessed performance on a holdout set, achieving an average ROC AUC of 0.85 closely matching the test set’s average ROC AUC of 0.85 (Supplementary Fig. 1). Supplementary Table 3 also compares performance metrics across five models, showing consistency between the test and holdout sets in ROC AUC (0.85), accuracy (0.92), PPA (0.48 vs. 0.50), PPV (0.06), F1-score (0.10), and NPA (0.93 vs. 0.92). This consistency in performance between test and holdout sets highlights the model’s robustness and supports its potential generalizability.

### ROS1 model performance stratified by specimen type

To stratify *ROS1* model performance by specimen type, we applied a classification model described in the “Methods” section to assign each WSI as either a biopsy or resection. On its independent evaluation set, this model achieved an AUC of 1.00, correctly classifying 108 out of 111 resections and all 82 biopsies. Given this high level of performance on external data, we considered the model reliable for use in assigning specimen type labels across our dataset. Based on its predictions, the cross-validation (CV) set included 18,370 biopsy cases and 9682 resections, while the holdout set comprised 3307 biopsies and 1655 resections.

For the test set, resection samples showed slightly lower ROC AUC (0.85) compared to biopsy samples (0.86) but exhibited higher sensitivity-related metrics. Specifically, PPA was 0.58 for resections versus 0.45 for biopsies, PPV was 0.07 vs. 0.05, and F1-score was 0.12 vs. 0.09. Accuracy and NPA were similar between the two specimen types: 0.91 vs. 0.92 for accuracy, and 0.92 for NPA in both groups.

For the holdout set, biopsy samples again achieved a slightly higher ROC AUC (0.86) compared to resections (0.84), while resections outperformed biopsies in most other metrics. PPA was 0.50 for resections versus 0.46 for biopsies, PPV was 0.08 vs. 0.05, and F1-score was 0.13 vs. 0.09. Accuracy was 0.92 for both groups, while NPA was 0.92 for resections and 0.93 for biopsies. A summary of these stratified metrics is provided in Table 2.

**Table 2:** Stratified performance metrics of the *ROS1* fusion prediction model on the test and holdout sets

	Test						Holdout					
	AUC	Acc	PPA	PPV	F1	NPA	AUC	Acc	PPA	PPV	F1	NPA
ALL	0.85	0.92	0.50	0.06	0.10	0.92	0.85	0.92	0.48	0.06	0.10	0.93
Bx	0.86	0.92	0.45	0.05	0.09	0.92	0.86	0.92	0.46	0.05	0.09	0.93
Rx	0.85	0.91	0.58	0.07	0.12	0.92	0.84	0.92	0.50	0.08	0.13	0.92

Results are reported for all cases (ALL), biopsy samples (Bx), and resection samples (Rx). Metrics include area under the ROC curve (AUC), accuracy (Acc), positive percent agreement (PPA), positive predictive value (PPV), F1-score (F1), and negative percent agreement (NPA). Values represent the average performance across five cross-validation folds. A positive class weight factor of 5 was used in the loss function.

*Bx* biopsy, *Rx* resection.

### Effect of positive weighting on *ROS1* fusion model

As indicated by our results presented in Table 3, adjusting the positive weight factor of the loss function had a notable impact on the model’s performance. For instance, when the positive weight (W) was set to 5, the model achieved a ROC AUC of 0.85 on both the test and holdout sets, with a PPA of 0.50 and 0.48, respectively, indicating a balanced ability to identify positive cases. However, as we increased the weight to 10, PPA improved to 0.58 for both sets, albeit with a slight drop in accuracy to 0.88 and 0.89, respectively, and a marginal decrease in PPV to 0.05.

**Table 3:** Impact of the positive weight (W) in the loss function on ROC AUC, accuracy (Acc), PPA, PPV, F1 score (F1), and NPA across test and holdout sets

	Test						Holdout					
W	AUC	Acc	PPA	PPV	F1	NPA	AUC	Acc	PPA	PPV	F1	NPA
**5**	0.85	0.92	0.50	0.06	0.10	0.92	0.85	0.92	0.48	0.06	0.10	0.93
**10**	0.85	0.88	0.58	0.05	0.09	0.89	0.85	0.89	0.58	0.05	0.09	0.89
**20**	0.86	0.84	0.72	0.04	0.08	0.84	0.85	0.84	0.68	0.04	0.07	0.84
**30**	0.85	0.79	0.79	0.04	0.07	0.79	0.85	0.79	0.72	0.03	0.06	0.79

These values represent the average across 5 folds for the ROS1 fusion models.

Further increasing the weight factor to 20 boosted the PPA to 0.72 on the test set and 0.68 on the holdout set, but this improvement came at the cost of accuracy and PPV, which dropped to 0.84 and 0.04, respectively. When the weight was set to 30, PPA reached 0.79 on the test set and 0.72 on the holdout set; however, this setting resulted in a significant decline in accuracy to 0.79 and a further decrease in PPV to 0.03 on the holdout set. Overall, while higher weight factors improved PPA, reducing the number of positive cases missed, they also led to an increase in false positives, impacting the model’s accuracy due to the prevalence of negatives.

**Fig. 3:**
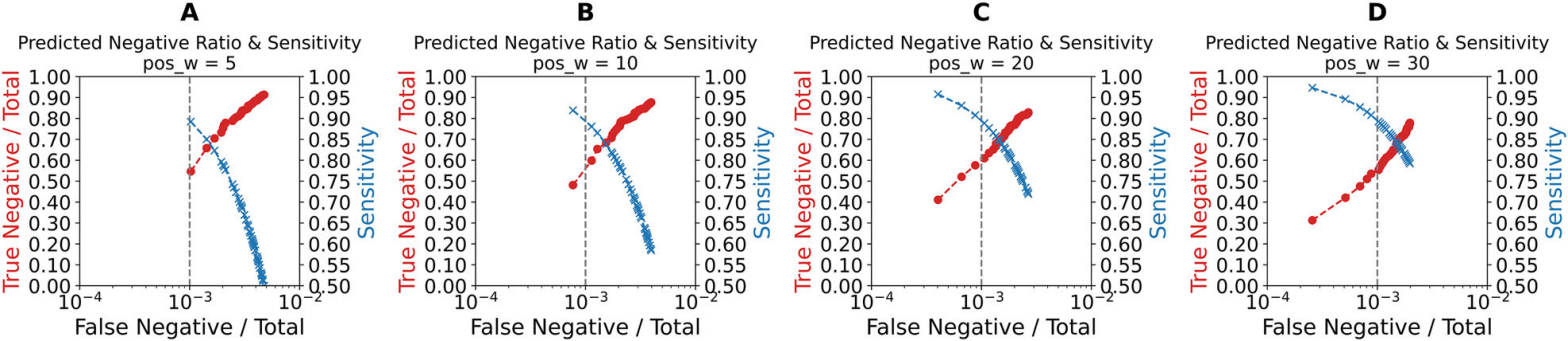
Impact of loss function weighting on ROS1 trade-off plots. **A**–**D** Trade-off plots for the *ROS1* test sets using different positive weights in the loss function: 5 (**A**), 10 (**B**), 20 (**C**), and 30 (**D**). The x-axis represents the false negative rate (false negatives divided by the total number of cases), while the y-axis represents the true negative rate (true negatives divided by the total cases) for the red plot, and PPA for the blue plot. Increasing the positive weight shifts the blue plot toward the top-left (indicating improved PPA and reduced false negatives) and the red plot toward the bottom-left (indicating reduced false negatives at the expense of lower true negatives).

We refer to the graphs in Fig. 3 as trade-off plots. Each panel in this figure shows the trade-off when different positive weights are applied in the loss function: 5, 10, 20, and 30. The x-axis represents the proportion of false negatives relative to the total cases, while the y-axis displays the true negative rate (true negatives divided by total cases) for the red curve and PPA for the blue curve. As the positive weight in the loss function increases, the blue curve shifts toward the top-left, reflecting improved PPA and reduced false negatives. However, this gain in PPA comes at the expense of a reduction in the true negative rate, as shown by the red curve shifting toward the bottom-left. This trade-off highlights how adjusting the positive weight can balance PPA and NPA based on the desired use case.

### *ALK* fusion model performance

**Fig. 4:**
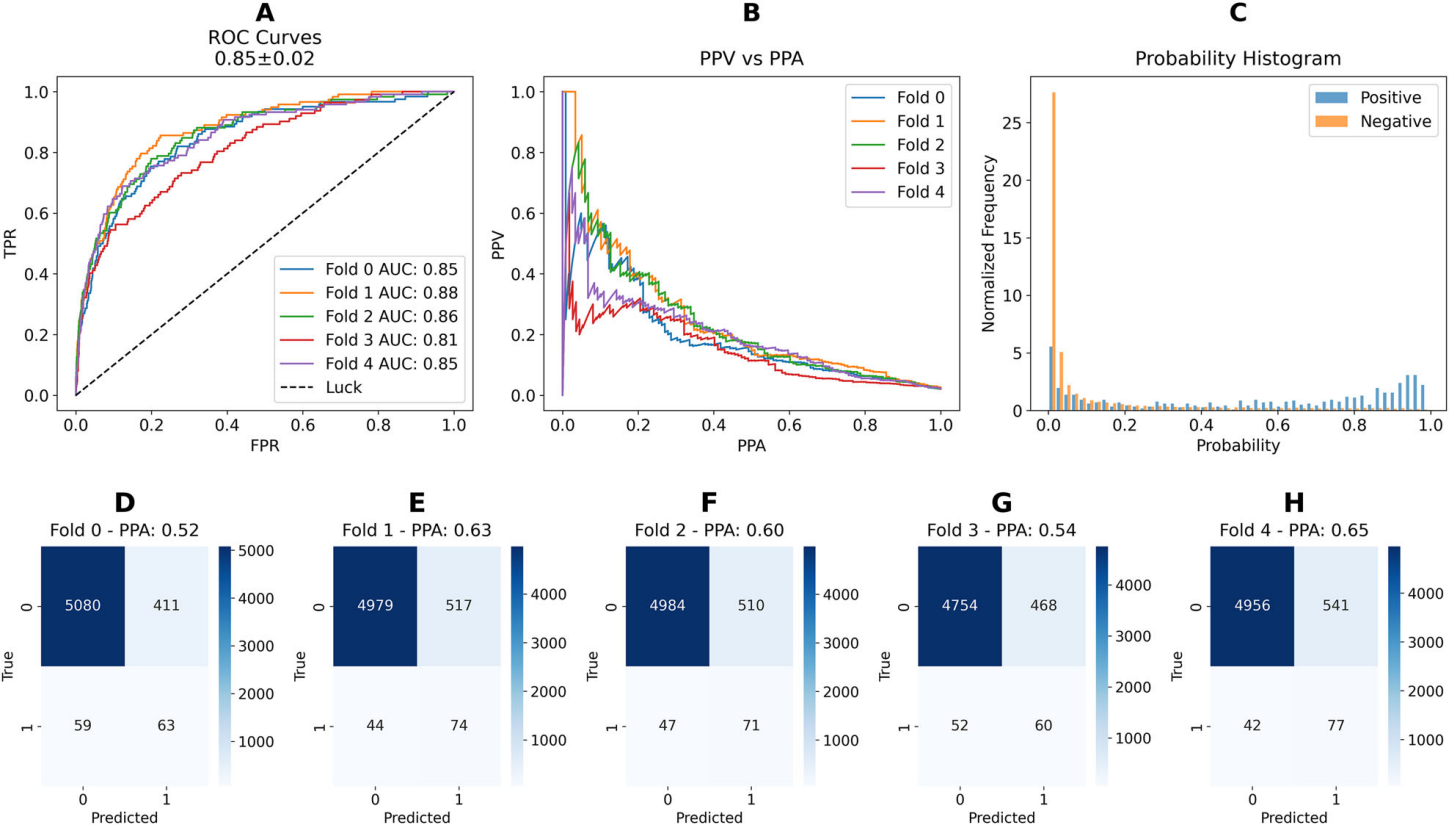
Performance plots for *ALK* prediction on the test sets using the train-finetune model. **A** Receiver Operating Characteristic (ROC) Curves: This panel displays the ROC curves for each cross-validation fold, with individual ROC AUC values listed in the legend. The average ROC AUC (0.85) ± standard deviation (0.02) across all folds is shown at the top of the plot. **B** Precision-Recall Curves: This panel presents the precision-recall curves for each fold. Precision corresponds to Positive Predictive Value (PPV), and recall corresponds to Positive Percent Agreement (PPA). **C** Normalized Probability Histogram: This panel shows the normalized histogram of predicted probabilities for the test set, distinguishing between positive and negative predictions. **D**–**H** Confusion Matrices for Individual Folds: These panels display the confusion matrices for different cross-validation folds (Fold 0 through Fold 4, respectively). In each matrix, true labels are compared to predicted labels. The Positive Percent Agreement (PPA) is indicated at the top of each confusion matrix.

Similarly to the *ROS1* fusion model, we also trained an *ALK* fusion model by initially training on the *RAN* dataset and subsequently fine-tuning it to predict *ALK* fusions. The results from the test set across different folds are displayed in Fig. 4. The ROC curves and corresponding ROC AUC values for each fold are shown in Fig. 4A, with the average ROC AUC displayed at the top of the plot. Figure 4B presents the precision-recall curves for the different folds (mean PR AUC: 0.20 ± 0.03, compared to 0.1 ± 0.03 for *ROS1*), while Fig. 4C displays a normalized histogram of predicted probability values. The confusion matrices for each fold are shown in Fig. 4D–H. Overall, these results indicate that although the *ALK* fusion model’s average ROC AUC is very similar to that of the *ROS1* fusion model, the *ALK* fusion model achieves a higher average PPA (0.57 vs. 0.50). This improvement is likely attributable to the larger number of positive *ALK* fusion cases compared to *ROS1* fusion positive cases.

To further validate the *ALK* fusion model, we evaluated its performance on a separate holdout set. The results were largely comparable to the test set, as shown in Supplementary Fig. 3 and Supplementary Table 4, with a reduction in PPA on the holdout set (0.54) compared to the test set (0.59). Performance metrics, including ROC AUC (0.84 holdout vs. 0.85 test), accuracy (0.90 for both datasets), PPV (0.11 holdout vs. 0.12 test), F1-score (0.19 holdout vs. 0.20 test), and NPA (0.91 for both datasets) consistent across both sets. This consistency suggests that the model’s performance generalizes well, and the small decrease in PPA does not significantly impact overall reliability.

#### ALK model performance stratified by specimen type

Similar to the *ROS1* analysis, we evaluated the *ALK* fusion model’s performance separately for biopsy and resection cases to explore whether specimen type affected prediction outcomes.

On the test set, both specimen types achieved similar AUC values—0.85 for biopsies and 0.86 for resections—but resection samples demonstrated higher sensitivity. Specifically, PPA was 0.56 for biopsies and 0.64 for resections, with corresponding PPVs of 0.11 and 0.14, and F1-scores of 0.19 and 0.23. Accuracy and NPA remained stable across groups (0.90–0.91 for accuracy and 0.91 for NPA).

In the holdout set, biopsy and resection AUCs were 0.84 and 0.85, respectively. As with the test set, resections achieved stronger performance on sensitivity-based metrics: PPA reached 0.60 compared to 0.50 for biopsies, PPV was 0.14 vs. 0.10, and F1-score was 0.23 vs. 0.16. Accuracy was 0.90 for both subgroups, and NPA was 0.91 for biopsies and 0.90 for resections. Stratified results for the *ALK* model are summarized in Table 4.

**Table 4:** Stratified performance metrics of the *ALK* fusion prediction model on the test and holdout sets

	Test						Holdout					
	AUC	Acc	PPA	PPV	F1	NPA	AUC	Acc	PPA	PPV	F1	NPA
ALL	0.85	0.90	0.59	0.12	0.20	0.91	0.84	0.90	0.54	0.11	0.19	0.91
Bx	0.85	0.90	0.56	0.11	0.19	0.91	0.84	0.90	0.50	0.10	0.16	0.91
Rx	0.86	0.91	0.64	0.14	0.23	0.91	0.85	0.90	0.60	0.14	0.23	0.90

Results are reported for all cases (ALL), biopsy samples (Bx), and resection samples (Rx). Metrics include area under the ROC curve (AUC), accuracy (Acc), positive percent agreement (PPA), positive predictive value (PPV), F1-score (F1), and negative percent agreement (NPA). Values represent the average performance across five cross-validation folds. A positive class weight factor of 5 was used in the loss function.

*Bx* biopsy, *Rx* resection.

### Effect of positive weighting on *ALK* fusion model

Similar to the *ROS1* fusion model, we examined the impact of increasing the positive weight of the loss function on various metrics for the *ALK* fusion model. Table 5 shows that, akin to the *ROS1* fusion model, a higher positive weight results in increased PPA but decreased accuracy and NPA on both the test and holdout sets. Specifically, as the positive weight increases from 5 to 30, PPA improves from 0.59 to 0.82 on the test set and from 0.54 to 0.81 on the holdout set. However, this improvement in PPA comes at the cost of accuracy, which decreases from 0.90 to 0.70 on both sets. PPV also drops from 0.12 to 0.06, and NPA falls from 0.91 to 0.69 on the test set, with similar trends observed on the holdout set. Despite these changes in PPA, PPV, accuracy, and NPA across different weight settings, the ROC AUC remains stable around 0.85. This highlights a limitation of ROC AUC as a metric in scenarios with imbalanced data, as it does not fully reflect the trade-offs or shifts in other performance metrics caused by changes in the positive weight.

**Table 5:** Impact of the positive weight (W) in the loss function on ROC AUC, accuracy (Acc), PPA, PPV, F1 score (F1), and NPA across test and holdout sets

	Test						Holdout					
W	AUC	Acc	PPA	PPV	F1	NPA	AUC	Acc	PPA	PPV	F1	NPA
**5**	0.85	0.90	0.59	0.12	0.20	0.91	0.84	0.90	0.54	0.11	0.19	0.91
**10**	0.85	0.82	0.72	0.08	0.15	0.83	0.85	0.82	0.69	0.08	0.15	0.83
**20**	0.85	0.74	0.8	0.06	0.12	0.73	0.85	0.74	0.78	0.06	0.12	0.74
**30**	0.85	0.70	0.82	0.06	0.10	0.69	0.84	0.70	0.81	0.06	0.11	0.69

These values represent the average across 5 folds for the ALK mode

**Fig. 5:**
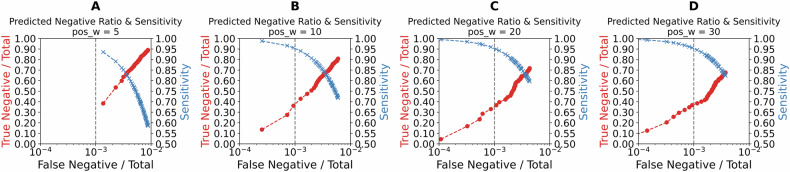
Impact of loss function weighting on *ALK* trade-off plots. Trade-off plots for the *ALK* test sets with varying positive weights in the loss function: 5 (**A**), 10 (**B**), 20 (**C**), and 30 (**D**). The x-axis shows the false negative rate (false negatives as a proportion of total cases), while the y-axis shows the true negative rate (true negatives as a proportion of total cases) for the red curve and PPA for the blue curve. As the positive weight increases, the blue curve shifts toward the top-left, reflecting enhanced PPA and fewer false negatives, while the red curve moves toward the bottom-left, reflecting a reduction in true negatives.

Similarly to Fig. 3 for the *ROS1* fusion model, Fig. 5A–D illustrates the trade-off for the *ALK* fusion model with varying positive weights (5, 10, 20, and 30) in the loss function. Figure 5B demonstrates that a PPA near 1 can be attained with a positive weight of 10 for the *ALK* fusion model, whereas for the *ROS1* fusion model, a similar PPA level requires a positive weight exceeding 30. However, this rapid increase in PPA for the *ALK* fusion model comes with a trade-off, as seen in the lower true negative rate across the red curve. This indicates that while the *ALK* fusion model achieves high PPA more efficiently, it does so at a greater cost to NPA, resulting in a lower proportion of true negatives overall. This difference emphasizes the model’s suitability for scenarios where PPA is prioritized over NPA.

### Model interpretability and visualization

**Fig. 6:**
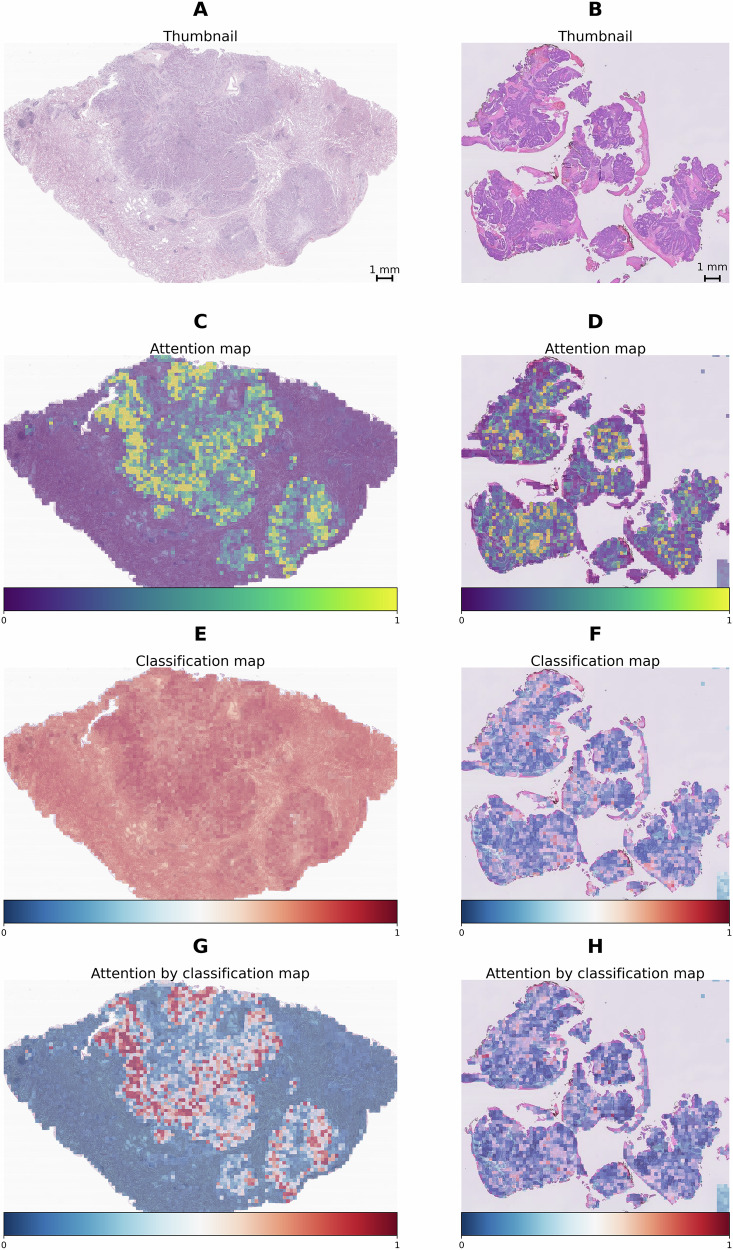
Visualization of model outputs for representative ROS1-positive and ROS1-negative cases. **A**, **C**, **E**, **G** *ROS1*-positive case; **B**, **D**, **F**, **H** *ROS1*-negative case. **A**, **B** Thumbnail images of the whole slide. **C**, **D** Predicted attention maps. **E**, **F** Predicted classification results. **G**, **H** Attention-by-classification maps. These visualizations illustrate how the model localizes key histologic features, focusing on informative regions to differentiate *ROS1*-positive from *ROS1*-negative samples.

The models we trained overcome the typical “black box” limitation of deep learning by generating attention and classification maps that highlight tissue regions contributing most to the final prediction. Figure 6 presents thumbnail, attention, and classification maps for a *ROS1*-positive case (A, C, E, G) and a *ROS1*-negative case (B, D, F, H). While some regions show high classification values, their impact on the final prediction is minimal if the corresponding attention values are low. High-attention areas primarily correspond to regions dense with invasive tumor cells, reflecting their biological relevance in predicting *ROS1* fusion status. These cells often exhibit distinct morphological and molecular features critical to biomarker prediction. Similarly, in the initial *RAN* classifier, attention maps frequently focused on tumor regions with high nuclear density and architectural complexity, suggesting these features may serve as general indicators of fusion positivity across *ROS1*, *ALK*, and *NTRK* subtypes (heatmaps not shown).

## Discussion

Deep learning models have significantly impacted the field of computer vision and histopathology image classification, excelling in tasks like cell segmentation^41–43^, image classification^44–46^, image retrieval^47–49^, stain normalization^50–52^, and biomarker prediction^53–55^. These models face challenges, particularly with the immense size of WSIs which exceed the processing capabilities of standard GPU memory. This issue necessitates segmenting WSIs into smaller, manageable tiles for batch processing.

Another hurdle in biomarker prediction is the absence of detailed labels for these tiles, often only having slide-level labels available. This challenge categorizes the learning process as weakly supervised, with Multiple Instance Learning (MIL) being a notable technique. MIL incorporates an attention mechanism that prioritizes the most informative regions within a slide, proving crucial for biomarker prediction using WSIs.

Vision transformers, inspired by transformers in natural language processing, have recently shown promise in this domain. They employ a robust attention mechanism to focus on key features in histopathology images, despite only having access to slide-level labels. This capability represents a significant advancement, closely aligning computational methods with clinical needs and facilitating the extraction of valuable insights from complex data.

Our study contributes to this growing field by using vision transformer models to predict *ROS1* and *ALK* gene fusions specifically in NSCLC. This cancer type was selected due to its prevalence, the clinical importance of identifying *ALK* and *ROS1* fusions for targeted therapy, and the need for rapid, cost-effective alternatives to traditional diagnostic methods such as FISH and IHC^13–16^. Despite their accepted utility, the cost and time involved in these traditional diagnostic techniques highlight the need for alternative approaches.

Building on existing research in biomarker detection via deep learning^17,21,56–59^, our model leverages a large dataset of 33,014 cases, including a wide range of specimen types such as resections and biopsies. These have been collected from 2020 to 2023 and scanned using both Philips and Leica systems, providing a rich and varied dataset that ensures our models are adaptable across different clinical settings.

A key innovation in our approach is the two-step training process where we initially train on a composite biomarker (*RAN*) and subsequently fine-tune the model for specific biomarkers like *ALK* or *ROS1* fusions. *ROS1*, *ALK*, and *NTRK* genes were selected for the combined label due to their similar biological pathways, as all three fusion proteins lead to constitutive activation of signaling pathways critical for tumorigenesis. *ROS1* fusion proteins lead to constitutive activation of their kinase domain, stimulating key signaling pathways: the RAS–MEK–ERK pathway (cell proliferation), the JAK–STAT3 pathway (cell growth and survival), and the PI3K–AKT–mTOR pathway (cell growth and survival)^60–62^. Similarly, *ALK* fusion proteins, such as EML4-*ALK*, persistently activate the *ALK* kinase domain, triggering the same pathways—RAS–MEK–ERK (proliferation), JAK–STAT3 (growth and survival), and PI3K–AKT–mTOR (growth and survival)^60,63,64^. Finally, *NTRK* gene fusions produce active TRK fusion proteins, activating the RAS–MEK–ERK (proliferation), PI3K–AKT–mTOR (growth and survival), and PLCγ (differentiation and survival) pathways^65,66^.

This method is particularly beneficial for *ROS1* fusions, where the number of positive cases is very limited, enhancing the model’s ability to detect these rare events more accurately. Figure 1 illustrates this, providing a comparative analysis that demonstrates the efficacy of our train-finetune approach. It highlights how this method improves model performance, especially in cases with fewer positive instances, such as *ROS1*. For *ALK*, however, the performance gain is more modest, which may be attributed to the larger number of *ALK* fusion-positive cases in our dataset (589 vs. 260 for *ROS1*; see Table 1), potentially buffering the impact of different training strategies.

In addition to overall performance, stratified analysis by specimen type (Tables 2 and 4) revealed consistent trends across both *ROS1* and *ALK* models. Although AUC and accuracy values were comparable between biopsies and resections, resection samples consistently achieved higher sensitivity-related metrics, including PPA, PPV, and F1-scores. This may be attributed to the greater tissue context and cellular diversity available in resection specimens, which can aid the model in identifying fusion-associated morphological features. These observations highlight the relevance of specimen type in interpreting model predictions and suggest that resection-based predictions may be more informative in certain clinical contexts.

Our use of trade-off plots in Figs. 3 and 5 provides a framework for healthcare administrators and researchers to explore the potential financial and clinical impact of using a pre-screening model. An accurate and cost-effective pre-screening model that confidently identifies a substantial portion of cases as negative would significantly reduce the number of cases requiring comprehensive NGS analysis^67,68^. While patients with NSCLC will undergo NGS profiling for comprehensive biomarker identification, the proposed model offers a targeted pre-screening mechanism to prioritize testing for *ALK* and *ROS1* fusions. This targeted approach reduces costs and accelerates clinical trial enrollment for therapies addressing these specific alterations. As an example, in a dataset of 1000 cases, if a model reliably identifies 500 as negative, only half would require NGS testing, resulting in considerable cost savings. However, it remains essential that any pre-screening model minimizes false negatives, as misclassifying positive cases could lead to missed opportunities for appropriate therapy.

The trade-off plots illustrate the balance between time and cost savings, and the risk of missing patients with actionable tumor-driving gene fusions. Therefore, maintaining high PPA is critical to minimize the risk of false negatives, as missing positive cases could lead to missed diagnostic opportunities and inappropriate therapy. Our two-step training approach not only improves the model’s accuracy but also enhances its clinical viability by reducing unnecessary testing and focusing resources on cases most likely to benefit from further analysis.

While these trade-off scenarios highlight the potential for cost-effective pre-screening, it is important to clarify that the primary intended role of our model is not to replace standard diagnostic tools such as IHC or RNA sequencing. Rather, we envision it as a decision-support tool that can assist clinicians and diagnostic labs in prioritizing and triaging cases for molecular testing. Especially in settings with limited resources or high sample volumes, the model can help direct attention to cases more likely to harbor actionable fusions, thereby optimizing the diagnostic workflow. Full integration into clinical decision-making would, of course, require rigorous external validation and alignment with existing diagnostic guidelines.

This study also addresses the bias typically seen in machine learning due to the uneven distribution of positive and negative cases. By adjusting the training dataset’s class distribution and modulating the loss function’s positive weight, we improved PPA without notably affecting the ROC AUC. As shown in Tables 3 and 5, these adjustments to the loss function’s weight factor had substantial effects on PPA, PPV, and accuracy, while the ROC AUC remains relatively stable across different weight factors, consistently around 0.85–0.86. This stability indicates that the models maintained a robust overall ability to distinguish between positive and negative cases.

While ROC AUC is an important metric for evaluating classification performance, it may not fully reflect the challenges posed by class imbalance. For instance, increasing the positive class weight improves PPA but may reduce overall accuracy. This occurs because the model is penalized more heavily for misclassifying positive samples, while misclassifications in the much larger negative class are penalized less. As a result, even slight increases in false positives—among the dominant negative class—can significantly reduce accuracy, which is defined as the proportion of all correctly classified cases. This trade-off highlights how the stability of ROC AUC can obscure meaningful shifts in performance, particularly in imbalanced datasets. Metrics such as PPA and PPV provide more granular insights into the model’s behavior on the minority class, which is essential for assessing clinical applicability. Precision-Recall (PR) curves, which focus on the performance of the positive class, may offer complementary insights to the ROC AUC and should be considered in future evaluations to provide a more comprehensive understanding of the model’s performance under imbalanced conditions.

The attention maps shown in Fig. 6 show that for both cases our model predominantly focuses on the tumor regions, as indicated by the shift from blue to yellow, with yellow denoting higher attention levels. In the classification map for the *ROS1* fusion negative case, several non-tumor areas are incorrectly marked with a higher likelihood of *ROS1* fusion positivity (shown in darker red). However, these areas receive little attention from our model, minimizing their impact on the final decision. This demonstrates the model’s effectiveness in distinguishing relevant from irrelevant areas in the analysis.

In addition to using MoCo-v3, we also integrated CTransPath features into our model training. Due to the unavailability of the source code for CTransPath, we were unable to fine-tune the model on our specific dataset and instead relied on the pretrained weights provided by the original developers. This approach resulted in ROC AUC values that were about 0.05 lower than those achieved with MoCo-v3, highlighting the critical role of both the feature extractor and the training dataset in achieving optimal model performance. To address the limitations imposed by reliance on pretrained weights, we are currently developing our own feature extraction pipeline, aiming to further improve model accuracy and suitability for our dataset.

While our findings demonstrate the promise of deep learning for fusion prediction, several limitations of this study should be acknowledged. One limitation relates to the use of feature extractors. Since completing the primary analyses for this study, several newer large-scale feature extractors have been introduced, such as PathCLIP^69^, Virchow^57^, Virchow 2^70^, UNI^59^, and CONCH^71^, which have demonstrated impressive performance across a variety of pathology tasks. While benchmarking these contemporary models is an important future direction, applying multiple extractors across a dataset of this scale (33,014 WSIs) presents substantial computational challenges. We acknowledge this as a limitation and encourage future studies to systematically evaluate the comparative performance of emerging feature extraction backbones in large-scale molecular prediction tasks.

A further consideration is the resolution of image tiles used for feature extraction. To improve computational efficiency, our feature extractor model was trained on 10× magnification tiles. While this choice offers scalability, it may result in the loss of finer cellular details that higher resolutions might capture. Incorporating higher-resolution tiles could potentially enhance performance, albeit with greater computational demands.

We also recognize the limitation posed by the low number of positive cases for *ROS1* and *ALK* fusion prediction, despite our dataset being significantly larger than those used in similar studies. This constraint reflects the inherently low prevalence of these alterations in NSCLC and is not unique to our dataset, but it nonetheless affects model training.

Another limitation is the absence of external independent validation. Although our dataset includes tissue samples submitted from hundreds of hospitals across the United States, all samples were processed and scanned within the same laboratory environment at Caris Life Sciences. As a result, while the data reflect broad clinical diversity in terms of origin, the imaging conditions are standardized. Consequently, the internal holdout set should not be interpreted as a fully independent external cohort, and future work should evaluate generalizability on data acquired under different scanning and processing conditions.

The study also lacks information on whether patients received prior treatments such as neoadjuvant therapy. While such treatments could potentially influence tissue morphology, our study focused on predicting fusion status as determined by RNA sequencing, which remains a stable ground truth regardless of treatment history. Moreover, because the dataset reflects routine clinical practice across a broad range of institutions, it likely includes both treated and untreated cases. As a result, the model was trained on morphologically heterogeneous samples, potentially capturing variation introduced by different clinical contexts. Nonetheless, the absence of explicit treatment metadata limits our ability to stratify performance by treatment history, and future work could explore this aspect in more detail.

Lastly, while the train-finetune approach demonstrates clear advantages in improving PPA for rare biomarkers like *ROS1* fusions, it introduces a trade-off in NPA. Pretraining on the composite *RAN* label, which groups *ALK* positive, *NTRK* positive, and *ROS1* positive cases together, biases the model towards identifying these fusions collectively. As a result, the train-finetune model shows an increased tendency to classify *ALK* positive or *NTRK* fusion positive cases as *ROS1* fusion positive, leading to a higher false positive rate compared to direct training. This trade-off is evident in our analysis, where the train-finetune model produced 252 false positives for *ALK*/*NTRK* fusion positive cases, compared to 103 in the direct model. Despite this, the primary objective of minimizing missed *ROS1* fusion positive cases is achieved, making the trade-off acceptable in clinical scenarios where PPA is paramount. This underscores the importance of tailoring model performance to the specific requirements of the use case, balancing the risks of false positives against the need to detect rare, actionable biomarkers.

In summary, by integrating deep learning into the diagnostic process, we aim to enhance the accuracy, efficiency, and cost-effectiveness of detecting *ALK* and *ROS1* fusions in NSCLC. This advancement holds the potential to streamline and improve oncology diagnostic tests, ultimately providing better outcomes for cancer patients.

## Methods

### Data acquisition and preprocessing

The dataset used in this study was derived from the Caris Life Sciences database, which comprises clinical specimens submitted from a wide range of healthcare institutions across the United States. These include hundreds of regional hospitals and medical centers, providing substantial diversity in both patient demographics and pre-analytic workflows. Among the 33,014 NSCLC patients available in this database, 306 were diagnosed as *ROS1*-fusion positive and 697 as *ALK*-fusion positive (Table 1). This heterogeneity and scale make the dataset well-suited for developing generalizable machine learning models.

WSIs were acquired at Caris Life Sciences laboratories using Leica and Philips scanners, introducing variability in imaging characteristics such as resolution, color profile, and compression. This variation mirrors real-world deployment conditions and further supports the robustness of the trained models.

*ROS1*, *ALK*, and *NTRK* fusion statuses were determined using a clinically validated next-generation sequencing (NGS)-based fusion assay performed by Caris Life Sciences. This assay detects gene fusions through targeted RNA sequencing and is certified under the Clinical Laboratory Improvement Amendments (CLIA) and accredited by the College of American Pathologists (CAP). This ensures that the fusion status labels used as ground truth for model training and evaluation are based on a gold-standard diagnostic method.

To prepare slides for model input, we applied a custom tissue preprocessing pipeline designed to identify diagnostically relevant tissue regions while excluding background and image artifacts. The process began with the extraction of low-resolution thumbnail images from each whole slide, which were used to identify and exclude blurry areas based on Laplacian edge detection smoothed by a Gaussian filter. Flat regions lacking sufficient texture were also removed using local averaging filters that detect uniform intensity. Small, isolated fragments and holes within tissue regions were handled through morphological operations that remove small objects and fill in small gaps to improve tissue mask continuity. To further refine the mask, enclosed low-density areas resembling fat were excluded, and dark artifacts such as printed slide labels were removed using intensity- and shape-based heuristics. These filtering steps were applied in sequence, each refining a shared binary mask to ensure only high-confidence, artifact-free tissue regions were retained. From the resulting tissue mask, we extracted non-overlapping tiles measuring 224 × 224 pixels at 10× magnification. These tiles were subsequently used in both the self-supervised learning phase and the downstream feature aggregation model.

### Experimental design

We designed a two-phase deep learning pipeline comprising: (1) self-supervised pretraining on histopathology tiles using MoCo-v3^34^, and (2) training of a vision transformer-based model to aggregate features for biomarker classification^32^. This two-stage approach allowed us to first learn generalizable visual representations from unannotated data, which were then used for biomarker-specific classification.

To begin, we set aside 15% of the entire dataset as an independent holdout set for final evaluation (Table 1). Because the tissue samples are collected from hundreds of submitting institutions across the United States, this random sampling strategy ensures that the holdout set also includes data from a diverse range of healthcare centers, supporting a robust evaluation of model generalizability. The remaining 85% constituted the CV set, which was used for both self-supervised pretraining and subsequent model training and evaluation. The MoCo-v3 model was trained exclusively on this CV set before any supervised learning or feature aggregation took place.

### Biopsy vs. resection label assignment

To assign specimen type labels (biopsy vs. resection) to WSIs, we used a dedicated classification model trained independently of the current study. This model was developed using a separate dataset comprising 1695 WSIs from diverse tissue lineages, including 836 biopsies and 859 resections.

Because the classification task relies on coarse morphological and contextual features rather than fine-grained cellular or molecular details, we used slide-level thumbnail images as input and adopted the VGG19 convolutional neural network architecture^72^, which is well suited for such image-level classification tasks. Thumbnail images were resized to 224 × 224 pixels, and the input pipeline included standard augmentation techniques such as flipping, rotation, and color jitter.

The model was trained using cross-entropy loss, the Adam optimizer (learning rate = 0.001), a batch size of 32, and 25 training epochs. This model was used to assign specimen type labels to all WSIs in our analysis pipeline.

### Feature extractor model

Self-supervised models, a subset of unsupervised learning approaches, leverage the data itself for learning. This approach is particularly advantageous in histopathology due to the abundance of unlabeled data and the high cost of expert annotations. In our study, we adopted this strategy to enable scalable representation learning without expert labels.

We utilized the MoCo-v3 model^34^ as our feature extractor. The choice of MoCo-v3 was influenced by its optimized implementation, the availability of open-source code, and its demonstrated success across a range of applications. MoCo-v3 uses a vision transformer backbone to encode image tiles into latent feature vectors. Its architecture includes two encoders: a query encoder, which is updated via gradient descent, and a key encoder, which is updated more slowly using momentum updates. This dual-encoder structure helps maintain consistent representations of negative samples across training steps. Its contrastive learning strategy involves matching randomly augmented crops of the same tile, which improves model stability and enhances robustness to common histopathology batch effects. The model processes each 224 × 224 pixel tile and outputs a 384-dimensional feature vector through the query encoder. This dimensionality follows the default configuration of MoCo-v3’s ViT-Small backbone, which was chosen to strike a balance between model expressiveness and computational efficiency, particularly important for large-scale feature extraction across tens of thousands of whole slide images.

For training, we selected 20 random tiles from each slide in the CV set. The model was initialized using pretrained MoCo-v3 weights from Wang et al.^73^, available on their GitHub repository^74^. Hyperparameters were set according to Supplementary Table 1.

**Fig. 7:**
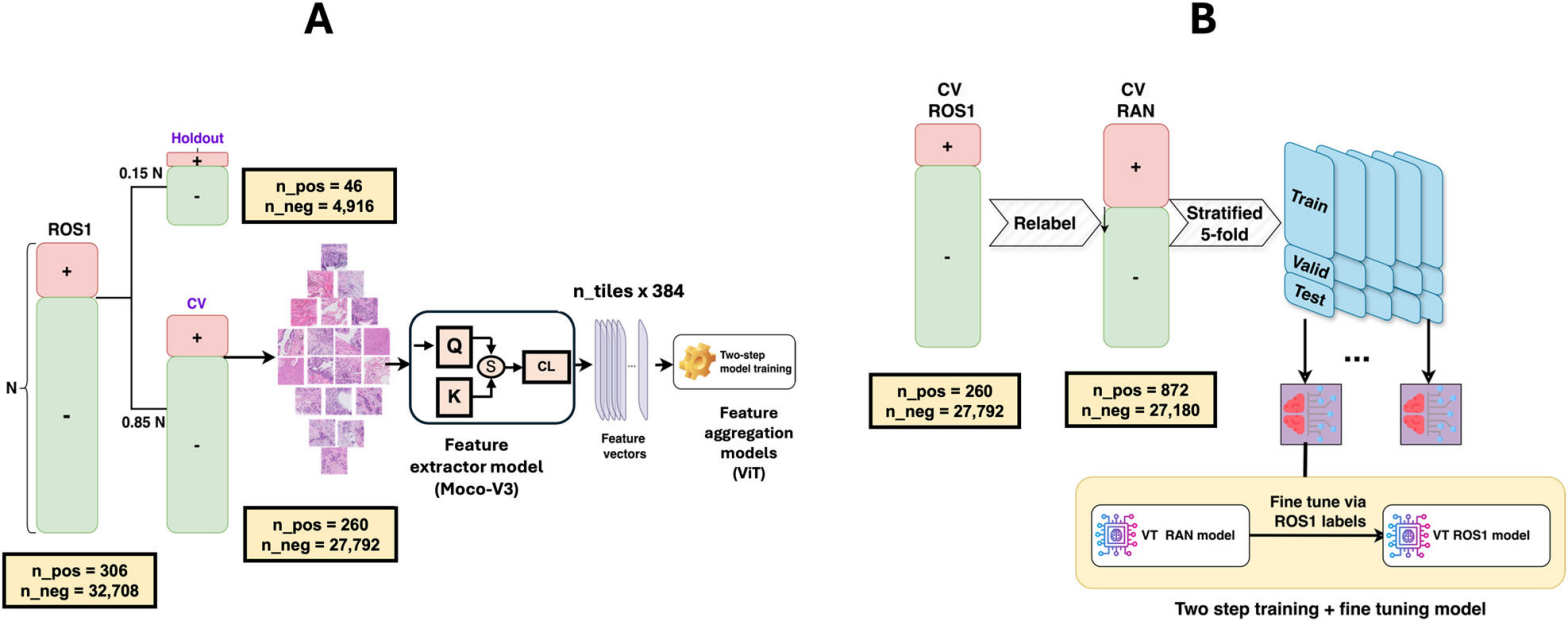
Overview of the self-supervised learning and two-step training framework for biomarker prediction. **A** Splitting the *ROS1* dataset into a cross-validation (CV) dataset and a holdout dataset, followed by training the MoCo-v3 model using the CV dataset. The trained MoCo-v3 model maps each tile to a feature vector of dimensions 1 × 384. These vectors are subsequently utilized to train a vision transformer-based model through a two-step process. Q query encoder, K key encoder, S similarity metric, CL contrastive loss, ViT vision transformer. **B** Dataset relabeling and two-step model training for *ROS1* prediction, with a similar methodology applied for *ALK* prediction. At each fold, the negative samples in the training data are randomly downsampled to a size five times that of the positive cases. The validation and test sets remain unchanged.

After training the MoCo-v3 model, features were extracted from each tile of every WSI. Each tile, sized 224 × 224 pixels, was processed by the model to produce a 384-dimensional feature vector. For each WSI, all tile-level feature vectors were stacked to form a matrix of size n_tiles × 384, where n_tiles denotes the number of tiles extracted from the WSI. This matrix organized the learned features for further downstream analysis. The full workflow for data splitting and MoCo-v3 training is illustrated in Fig. 7A. As also shown in this figure, the extracted features are used in the subsequent training of a vision transformer-based feature aggregation model, described in the next section.

### Feature aggregation model

To generate slide-level predictions from tile-level features, we employed a vision transformer-based aggregation model. This model learns to integrate information across multiple image regions, attending to the most relevant tiles to predict molecular status. Because transformer-based architectures are well-suited for capturing contextual dependencies, they are ideal for modeling spatial relationships across histopathology tiles.

The aggregation model architecture begins with a projection layer that transforms the n_tiles × 384 feature vectors into n_tiles × 512 vectors. These projected vectors, along with a classification token (CLS), are passed through two transformer layers, each with 8 attention heads. The output is then processed by a multi-layer perceptron (MLP) layer, which generates a prediction for the specific biomarker^32^.

Initially, we directly trained a model on the *ROS1* dataset; however, we encountered a significant limitation due to the small number of *ROS1*-positive cases, which is suboptimal for training deep learning models. As noted in the “Experimental Design” section, 15% of the data was set aside as a holdout set, leaving 260 *ROS1*-positive cases in the CV set out of the total 306 available (Table 1). To overcome this challenge, we relabeled our CV dataset using a new combined label termed *RAN* (*ROS1*–*ALK*–*NTRK*). These three genes were selected due to their shared biological role in activating overlapping oncogenic signaling pathways (detailed rationale provided in the Discussion). A sample was classified as *RAN*-positive if it contained any gene fusion of *ROS1*, *ALK*, or *NTRK*, and *RAN*-negative if none of these gene fusions were present. This relabeling strategy increased the number of positive cases from 260 (*ROS1*) to 872 (*RAN*). We first trained a model to predict *RAN* status and subsequently fine-tuned it to predict *ROS1* fusions.

### Cross-validation design and training

As illustrated in Fig. 7B, we constructed five independent stratified splits from the CV set based on the *RAN* label. Each split consisted of 60% for training, 20% for validation, and 20% for testing. The model was first trained to predict *RAN* status on these splits and then fine-tuned to predict *ROS1* status using the same splits, ensuring no contamination between the first and second training phases.

This three-way train/validation/test split approach enables unbiased hyperparameter tuning, unlike simpler train/validation setups where the final performance may be indirectly influenced by the validation set. In the training set, the negative class was randomly downsampled to be five times the size of the positive class, and the loss function assigned a weight of 5 to the positive class. Multiple sampling and weighting configurations were tested, and the one achieving the best trade-off between PPA and NPA on the validation set was selected.

In addition to evaluating performance on the test set of each split, we also assessed each trained model on the 15% holdout set that was reserved prior to pretraining the feature extractor. This confirmed that the final results were not influenced by any data seen during self-supervised training.

### Heatmap generation and model interpretation

To interpret model predictions and visualize spatial patterns, we generated heatmaps using the framework developed by Wagner et al.^32^. Attention maps indicate which regions of a WSI had the greatest influence on the final prediction. These maps are derived from the self-attention mechanism of the transformer-based aggregation model, where each tile’s feature vector is treated analogously to a token embedding in natural language models. For each tile, the model computes query, key, and value vectors. Attention weights are obtained via scaled dot-product between queries and keys, followed by softmax normalization. These weights determine the contribution of each tile (value) to the final slide-level representation. To quantify this influence, we applied attention rollout across all transformer layers, which recursively aggregates attention scores and accounts for residual connections^75^.

In parallel, we produced classification maps showing the predicted class for each tile based on its feature representation. Although the model was trained to predict slide-level fusion status, we approximated tile-level classification scores by passing each tile’s feature vector independently through the trained classifier. While this approximation is not explicitly trained for tile-wise prediction, it offers insight into which regions exhibit biomarker-associated morphology.

Finally, we generated attention-weighted classification maps by element-wise multiplying tile-level classification scores with their corresponding attention scores. This highlights tiles that were both predictive and influential, providing interpretable spatial context to support model transparency.

## Supplementary information

Supplementary_materials

## Data Availability

The data and datasets utilized in this study are available from the corresponding author upon reasonable request. Deidentified data are owned by Caris Life Sciences and cannot be publicly shared because of the data usage agreement. Qualified researchers can apply for access to these data by contacting Joanne Xiu, PhD (jxiu@carisls.com) and signing a data usage agreement.

## Code availability

The code was developed based on the open-source project HistoBistro (https://github.com/peng-lab/HistoBistro). Detailed implementation is provided in the “Methods” section of the manuscript. The code will be made available for replication and verification purposes from the corresponding author upon signing a code usage agreement and subject to company approval.

## References

[1] American Cancer Society. (n.d.). Lung Cancer Survival Rates. Cancer.org. https://www.cancer.org/cancer/types/lung-cancer/detection-diagnosis-staging/survival-rates.html

[2] American Cancer Society. (n.d.). Key Statistics for Lung Cancer. Cancer.org. https://www.cancer.org/cancer/types/lung-cancer/about/key-statistics.html

[3] Minna, J. D., Roth, J. A. & Gazdar, A. F. Focus on lung cancer. *Cancer Cell* **1**, 49–52 (2002). 12086887 10.1016/s1535-6108(02)00027-2

[4] Lemjabbar-Alaoui, H., Hassan, O. U., Yang, Y. W. & Buchanan, P. Lung cancer: biology and treatment options. *Biochim Biophys. Acta* **1856**, 189–210 (2015). 26297204 10.1016/j.bbcan.2015.08.002 PMC4663145

[5] Marinelli, D. et al. Non-small-cell lung cancer: how to manage *ALK*-, *ROS1*- and *NTRK*-rearranged disease. *Drugs Context* **11**, 2022-3-1. 10.7573/dic.2022-3-1 (2022). 10.7573/dic.2022-3-1 PMC9576009 36303600

[6] Stransky, N., Cerami, E., Schalm, S., Kim, J. L. & Lengauer, C. The landscape of kinase fusions in cancer. *Nat. Commun.* **5**, 4846 (2014). 25204415 10.1038/ncomms5846 PMC4175590

[7] Bergethon, K. et al. *ROS1* rearrangements define a unique molecular class of lung cancers. *J. Clin. Oncol.* **30**, 863–870 (2012). 22215748 10.1200/JCO.2011.35.6345 PMC3295572

[8] Pan, Y. et al. *ALK*, *ROS1* and RET fusions in 1139 lung adenocarcinomas: a comprehensive study of common and fusion pattern-specific clinicopathologic, histologic and cytologic features. *Lung Cancer* **84**, 121–126 (2014). 24629636 10.1016/j.lungcan.2014.02.007

[9] Kohno, T. et al. Beyond *ALK*-RET, *ROS1* and other oncogene fusions in lung cancer. *Transl. Lung Cancer Res.* **4**, 156–164 (2015). 25870798 10.3978/j.issn.2218-6751.2014.11.11 PMC4384213

[10] Sgambato, A., Casaluce, F., Maione, P. & Gridelli, C. Targeted therapies in non-small cell lung cancer: a focus on *ALK*/*ROS1* tyrosine kinase inhibitors. *Expert Rev. Anticancer Ther.* **18**, 71–80 (2018). 29187012 10.1080/14737140.2018.1412260

[11] Remon, J., Pignataro, D., Novello, S. & Passiglia, F. Current treatment and future challenges in *ROS1*- and *ALK*-rearranged advanced non-small cell lung cancer. *Cancer Treat. Rev.* **95**, 102178 (2021). 33743408 10.1016/j.ctrv.2021.102178

[12] Ou, S. H., Tan, J., Yen, Y. & Soo, R. A. *ROS1* as a ‘druggable’ receptor tyrosine kinase: lessons learned from inhibiting the *ALK* pathway. *Expert Rev. Anticancer Ther.* **12**, 447–456 (2012). 22500682 10.1586/era.12.17

[13] Niu, X., Chuang, J. C., Berry, G. J. & Wakelee, H. A. Anaplastic lymphoma kinase testing: IHC vs. FISH vs. NGS. *Curr. Treat. Options Oncol.* **18**, 71 (2017). 29143897 10.1007/s11864-017-0513-x

[14] Lazzari, C. et al. Next generation sequencing in non-small cell lung cancer: pitfalls and opportunities. *Diagnostics* **10**, 1092 (2020). 33333743 10.3390/diagnostics10121092 PMC7765222

[15] Bubendorf, L. et al. Testing for *ROS1* in non-small cell lung cancer: a review with recommendations. *Virchows Arch.* **469**, 489–503 (2016). 27535289 10.1007/s00428-016-2000-3 PMC5082594

[16] Scattone, A. et al. Discordance between FISH, IHC, and NGS analysis of *ALK* status in advanced non–small cell lung cancer (NSCLC): a brief report of 7 cases. *Transl. Oncol.* **12**, 389–395 (2019). 30529852 10.1016/j.tranon.2018.11.006 PMC6280637

[17] Echle, A. et al. Deep learning in cancer pathology: a new generation of clinical biomarkers. *Br. J. Cancer* **124**, 686–696 (2021). 33204028 10.1038/s41416-020-01122-x PMC7884739

[18] Gamble, P. et al. Determining breast cancer biomarker status and associated morphological features using deep learning. *Commun. Med.* **1**, 14 (2021). 35602213 10.1038/s43856-021-00013-3 PMC9037318

[19] Li, F. et al. Deep learning-based predictive biomarker of pathological complete response to neoadjuvant chemotherapy from histological images in breast cancer. *J. Transl. Med.* **19**, 348 (2021). 34399795 10.1186/s12967-021-03020-z PMC8365907

[20] Arslan, S. et al. A systematic pan-cancer study on deep learning-based prediction of multi-omic biomarkers from routine pathology images. *Commun. Med.* **4**, 48 (2024). 38491101 10.1038/s43856-024-00471-5 PMC10942985

[21] van der Laak, J., Litjens, G. & Ciompi, F. Deep learning in histopathology: the path to the clinic. *Nat. Med.* **27**, 775–784 (2021). 33990804 10.1038/s41591-021-01343-4

[22] Lancellotti, C. et al. Artificial intelligence & tissue biomarkers: advantages, risks and perspectives for pathology. *Cells* **10**, 787 (2021). 33918173 10.3390/cells10040787 PMC8066881

[23] Unger, M. & Kather, J. N. Deep learning in cancer genomics and histopathology. *Genome Med.* **16**, 44 (2024). 38539231 10.1186/s13073-024-01315-6 PMC10976780

[24] Wang, C.-W. et al. Deep learning to assess microsatellite instability directly from histopathological whole slide images in endometrial cancer. *NPJ Digit. Med.* **7**, 143 (2024). 38811811 10.1038/s41746-024-01131-7 PMC11137095

[25] Zhao, Y. et al. Deep learning using histological images for gene mutation prediction in lung cancer: a multicentre retrospective study. *Lancet Oncol.* **26**, 136–146 (2025). 39653054 10.1016/S1470-2045(24)00599-0

[26] Kong, X. et al. A deep-learning model for predicting tyrosine kinase inhibitor response from histology in gastrointestinal stromal tumor. *J. Pathol.*10.1002/path.6329 (2025). 10.1002/path.6399 39950223

[27] Dundar, et al. A multiple instance learning approach toward optimal classification of pathology slides. In *Proc. 20th International Conference on Pattern Recognition, Istanbul, Turkey* 2732–2735, 10.1109/ICPR.2010.669 (2010).

[28] Gadermayr, M. & Tschuchnig, M. Multiple instance learning for digital pathology: a review of the state-of-the-art, limitations & future potential. *Comput. Med. Imaging Graph.* **112**, 102337 (2024). 38228020 10.1016/j.compmedimag.2024.102337

[29] Sandarenu, P. et al. Survival prediction in triple negative breast cancer using multiple instance learning of histopathological images. *Sci. Rep.* **12**, 14527 (2022). 36008541 10.1038/s41598-022-18647-1 PMC9411153

[30] Jin, D. et al. Teacher-student collaborated multiple instance learning for pan-cancer PDL1 expression prediction from histopathology slides. *Nat. Commun.* **15**, 3063 (2024). 38594278 10.1038/s41467-024-46764-0 PMC11004138

[31] Couture, H. D. Deep learning-based prediction of molecular tumor biomarkers from H&E: a practical review. *J. Pers. Med.* **12**, 2022 (2022). 36556243 10.3390/jpm12122022 PMC9784641

[32] Wagner, S. J. et al. Transformer-based biomarker prediction from colorectal cancer histology: a large-scale multicentric study. *Cancer Cell* **41**, 1650–1661.e4 (2023). 37652006 10.1016/j.ccell.2023.08.002 PMC10507381

[33] Lupo, C. et al. Machine learning in computational pathology through self-supervised learning and vision transformers. In *Advanced Studies in Complex Systems: Theory and Applications, Artificial Intelligence for Medicine* 25–35 (Academic Press, 2024) ISBN 9780443136719.

[34] Chen, X., Xie, S. & He, K. An empirical study of training self-supervised visual transformers. In *Proc. IEEE/CVF International Conference on Computer Vision.* 9640–9649 (IEEE, 2021).

[35] Wang, X. et al. Transformer-based unsupervised contrastive learning for histopathological image classification. *Med. Image Anal.* **81**, 102559 (2022). 35952419 10.1016/j.media.2022.102559

[36] Mayer, C. et al. Direct identification of *ALK* and *ROS1* fusions in non-small cell lung cancer from hematoxylin and eosin-stained slides using deep learning algorithms. *Mod. Pathol.* **35**, 1882–1887 (2022). 36057739 10.1038/s41379-022-01141-4 PMC9708557

[37] Terada, Y. et al. Artificial intelligence–powered prediction of *ALK* gene rearrangement in patients with non–small-cell lung cancer. *JCO Clin. Cancer Inf.* **6**, e2200070 (2022). 10.1200/CCI.22.00070 36162012

[38] Coudray, N. et al. Classification and mutation prediction from non–small cell lung cancer histopathology images using deep learning. *Nat. Med.* **24**, 1559–1567 (2018). 30224757 10.1038/s41591-018-0177-5 PMC9847512

[39] Tan, X. et al. Predicting EGFR mutation, *ALK* rearrangement, and uncommon EGFR mutation in NSCLC patients by driverless artificial intelligence: a cohort study. *Respir. Res.* **23**, 132 (2022). 35624472 10.1186/s12931-022-02053-2 PMC9145462

[40] Mandrekar, J. N. Receiver operating characteristic curve in diagnostic test assessment. *J. Thorac. Oncol.* **5**, 1315–1316 (2010). 20736804 10.1097/JTO.0b013e3181ec173d

[41] Kumar, N. et al. A dataset and a technique for generalized nuclear segmentation for computational pathology. *IEEE Trans. Med. Imaging* **36**, 1550–1560 (2017). 28287963 10.1109/TMI.2017.2677499

[42] Li, L., He, K., Zhu, X., Gou, F. & Wu, J. A pathology image segmentation framework based on deblurring and region proxy in medical decision-making system. *Biomed. Signal Process. Control* **95**, 106439 (2024).

[43] Basu, A., Senapati, P., Deb, M., Rai, R. & Dhal, K. G. A survey on recent trends in deep learning for nucleus segmentation from histopathology images. *Evol. Syst.* **15**, 203–248 (2024). 10.1007/s12530-023-09491-3 PMC9987406 38625364

[44] Zhu, C., Chen, W., Peng, T., Wang, Y. & Jin, M. Hard sample aware noise robust learning for histopathology image classification. *IEEE Trans. Med. Imaging* **41**, 881–894 (2021). 10.1109/TMI.2021.3125459 34735341

[45] Asadi-Aghbolaghi, M. et al. Learning generalizable AI models for multi-center histopathology image classification. *NPJ Precis. Oncol.* **8**, 151 (2024). 39030380 10.1038/s41698-024-00652-4 PMC11271637

[46] Balasubramanian, A. A. et al. Ensemble deep learning-based image classification for breast cancer subtype and invasiveness diagnosis from whole slide image histopathology. *Cancers* **16**, 2222 (2024). 38927927 10.3390/cancers16122222 PMC11201924

[47] Mohammad Alizadeh, S., Sadegh Helfroush, M. & Müller, H. A novel Siamese deep hashing model for histopathology image retrieval. *Expert Syst. Appl.* **225**, 120169 (2023).

[48] Hu, D. et al. Histopathology language-image representation learning for fine-grained digital pathology cross-modal retrieval. *Med. Image Anal.* **95**, 103163 (2024). 38626665 10.1016/j.media.2024.103163

[49] Saeidi, N. et al. Breast histopathology image retrieval by attention-based adversarially regularized variational graph autoencoder with contrastive learning-based feature extraction. arXiv preprint. 10.48550/arXiv.2405.04211 (2024).

[50] Kang, H. et al. StainNet: A fast and robust stain normalization network. *Front. Med.* **8**, 746307 (2021). 10.3389/fmed.2021.746307 PMC8602577 34805215

[51] Hoque, M. Z., Keskinarkaus, A., Nyberg, P. & Seppänen, T. Stain normalization methods for histopathology image analysis: a comprehensive review and experimental comparison. *Inf. Fusion* **102**, 101997 (2024).

[52] Ye, H. et al. Stain-adaptive self-supervised learning for histopathology image analysis. *Pattern Recognit.* **161**, 111242 (2025).

[53] Mandair, D., Reis-Filho, J. S. & Ashworth, A. Biological insights and novel biomarker discovery through deep learning approaches in breast cancer histopathology. *NPJ Breast Cancer* **9**, 21 (2023). 37024522 10.1038/s41523-023-00518-1 PMC10079681

[54] El Nahhas, O. et al. From whole-slide image to biomarker prediction: End-to-end weakly supervised deep learning in computational pathology. *Nat. Protoc.* **20**, 293–316 (2025). 39285224 10.1038/s41596-024-01047-2

[55] Filiot, A. et al. Phikon-v2, a large and public feature extractor for biomarker prediction. arXiv preprint. 10.48550/arXiv.2409.09173 (2024).

[56] Vorontsov, E. et al. Virchow: a million-slide digital pathology foundation model. arXiv preprint. 10.48550/arXiv.2309.07778 (2023).

[57] Vorontsov, E. et al. A foundation model for clinical-grade computational pathology and rare cancers detection. *Nat. Med.* **30**, 2924–2935 (2024). 39039250 10.1038/s41591-024-03141-0 PMC11485232

[58] Pai, S. et al. Foundation model for cancer imaging biomarkers. *Nat. Mach. Intell.* **6**, 354–367 (2024). 38523679 10.1038/s42256-024-00807-9 PMC10957482

[59] Chen, R. J. et al. Towards a general-purpose foundation model for computational pathology. *Nat. Med.* **30**, 850–862 (2024). 38504018 10.1038/s41591-024-02857-3 PMC11403354

[60] Rosas, D. et al. Molecular targeting in non-small cell lung cancer: advances in EGFR, *ROS1*, *ALK*, and MET pathways. *J. Oncol. Res. Ther.* **9**, 10214 (2024).

[61] D’Angelo, A. et al. Focus on *ROS1*-positive non-small cell lung cancer (NSCLC): crizotinib, resistance mechanisms and the newer generation of targeted therapies. *Cancers* **12**, E3293 (2020). 10.3390/cancers12113293 PMC7694780 33172113

[62] Davies, K. D. & Doebele, R. C. Molecular pathways: *ROS1* fusion proteins in cancer. *Clin. Cancer Res.* **19**, 4040–4045 (2013). 23719267 10.1158/1078-0432.CCR-12-2851 PMC3732549

[63] Ducray, S. P., Natarajan, K., Garland, G. D., Turner, S. D. & Egger, G. The transcriptional roles of *ALK* fusion proteins in tumorigenesis. *Cancers* **11**, 1074 (2019). 31366041 10.3390/cancers11081074 PMC6721376

[64] Martorana, F. et al. AKT inhibitors: new weapons in the fight against breast cancer?. *Front. Pharm.* **12**, 662232 (2021). 10.3389/fphar.2021.662232 PMC8118639 33995085

[65] Amatu, A., Sartore-Bianchi, A. & Siena, S. *NTRK* gene fusions as novel targets of cancer therapy across multiple tumour types. *ESMO Open* **1**, e000023 (2016). 27843590 10.1136/esmoopen-2015-000023 PMC5070277

[66] Kheder, E. S. & Hong, D. S. Emerging targeted therapy for tumors with *NTRK* fusion proteins. *Clin. Cancer Res.* **24**, 5807–5814 (2018). 29986850 10.1158/1078-0432.CCR-18-1156

[67] Echle, A. et al. Artificial intelligence for detection of microsatellite instability in colorectal cancer—a multicentric analysis of a pre-screening tool for clinical application. *ESMO Open* **7**, 100400 (2022). 35247870 10.1016/j.esmoop.2022.100400 PMC9058894

[68] Wang, Y. K. et al. Screen them all: high-throughput pan-cancer genetic and phenotypic biomarker screening from H&E whole slide images. arXiv preprint. 10.48550/arXiv.2408.09554 (2024).

[69] He, F. et al. PathCLIP: Detection of genes and gene relations from biological pathway figures through image-text contrastive learning. *IEEE J. Biomed. Health Inf.* **28**, 5007–5019 (2024). 10.1109/JBHI.2024.3383610 PMC11363067 38568768

[70] Zimmermann, et al. Virchow2: Scaling self-supervised mixed magnification models in pathology. arXiv preprint. 10.48550/arXiv.2408.00738 (2024).

[71] Lu, M. Y. et al. A visual-language foundation model for computational pathology. *Nat. Med.* **30**, 863–874 (2024). 38504017 10.1038/s41591-024-02856-4 PMC11384335

[72] Simonyan, K. & Zisserman, A. Very deep convolutional networks for large-scale image recognition. arXiv preprint. https://arxiv.org/abs/1409.1556 (2014).

[73] Wang, X. et al. TransPath: transformer-based self-supervised learning for histopathological image classification. In *Proceedings of the International Conference on Medical Image Computing and Computer-Assisted Intervention* 186–195 (Springer, 2021).

[74] Wang, X. et al. (n.d.). TransPath. GitHub repository. Available at: https://github.com/Xiyue-Wang/TransPath.

[75] Abnar, S. & Zuidema, W. Quantifying attention flow in transformers. In *Proceedings of the 58th Annual Meeting of the Association for Computational Linguistics* 4190–4197 (Association for Computational Linguistics, 2020).

