# Supplementary material for: Predicting ROS1 and ALK fusions in NSCLC from H&E slides with a two-step vision transformer approach
Source: NPJ Precis Oncol. 2025 Jul 30;9:266. doi: 10.1038/s41698-025-01037-x (PMC12311174; doi:10.1038/s41698-025-01037-x)
Supplement: Supplementary file 1 — Supplementary_materials [file 41698_2025_1037_MOESM1_ESM.pdf]

Supplementary information

MoCo-v3 hyperparameters:

Supplementary Table 1*: The hyperparameters and respective values for the MoCo-v3 model trained in this work.*

Hyperparameter	Value
Number of epochs	300
Number of warmup epochs	20
Batch size	256
Learning rate	1.5e-4
Weight decay	0.1

Aggregator model hyperparameters:

Supplementary Table 1: The hyperparameters of the vision transformer based aggregator model.

Hyperparameter	Value
lr	1e-05 (parent model) / 1e-06 (fine-tuned model)
wd	1e-05 (parent model) / 1e-06 (fine-tuned model)
num_epochs	40
max_num_tiles_per_slide	1000
Batch size	1
Criterion	BCEWithLogitsLoss
Optimizer	AdamW

ROS1 fusion model performance on hold out set

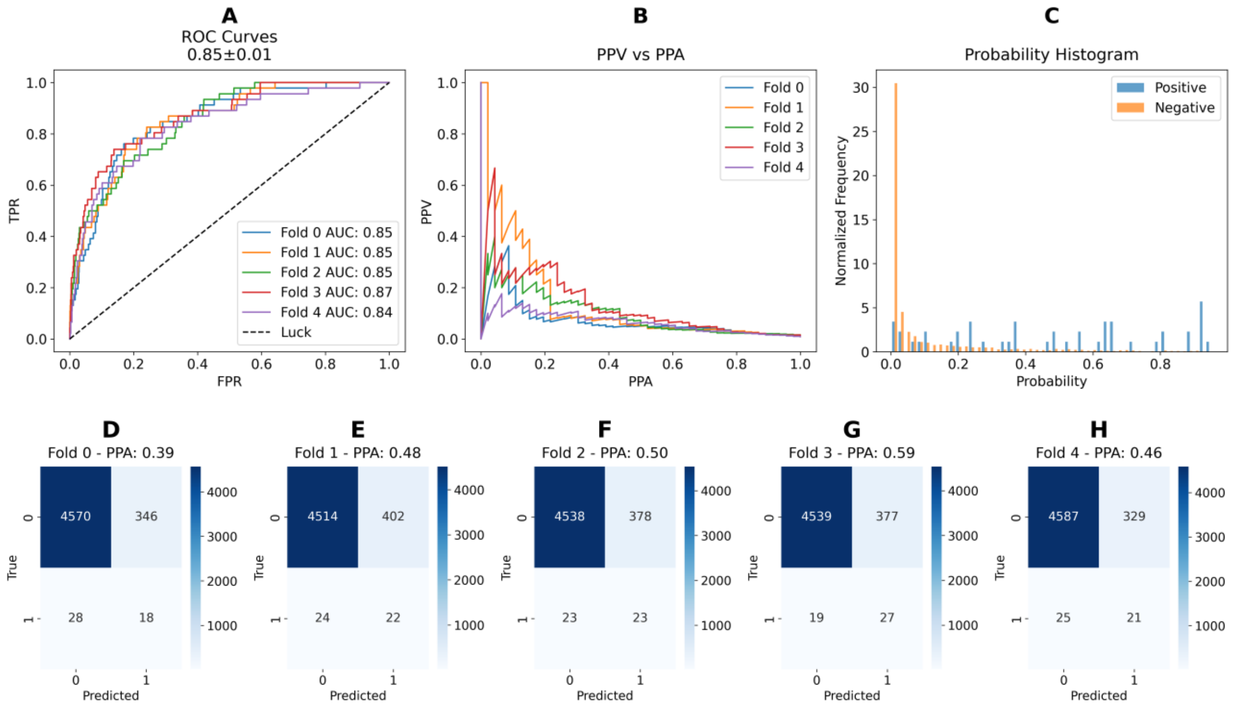

Supplementary Figure 1: Performance plots for ROS1 on the hold sets using the train-finetune model. (A) shows the ROC curve, with ROC AUC values for each fold included in the legend and the average ± standard deviation ROC AUC displayed at the top of the plot. (B) presents the precision-recall curve, and (C) shows the normalized histogram of the predicted probabilities. Panels (D–H) display the confusion matrices for different folds, where true labels are compared to predicted labels, and the true positive rate (PPA) is indicated at the top of each plot.

Supplementary Table 3: Comparison of the performance of the 5 trained ROS1 fusion models on test and holdout sets.

	**Fold 0**		**Fold 1**		**Fold 2**		**Fold 3**		**Fold 4**		**Average**	
	Test	***Holdout***	***Test***	***Holdout***	***Test***	***Holdout***	***Test***	***Holdout***	***Test***	***Holdout***	***Test***	***Holdout***
**AUC**	0.84	0.85	0.86	0.85	0.89	0.85	0.86	0.87	0.82	0.84	0.85	0.85
**Accuracy**	0.92	0.92	0.92	0.91	0.91	0.92	0.92	0.92	0.93	0.93	0.92	0.92
**PPA**	0.45	0.39	0.47	0.48	0.53	0.5	0.54	0.59	0.5	0.46	0.5	0.48
**PPV**	0.05	0.05	0.05	0.05	0.05	0.06	0.07	0.07	0.06	0.06	0.06	0.06
**F1-score**	0.09	0.09	0.1	0.09	0.01	0.1	0.12	0.12	0.11	0.11	0.10	0.10
**NPA**	0.92	0.93	0.92	0.92	0.91	0.92	0.92	0.92	0.93	0.93	0.92	0.93

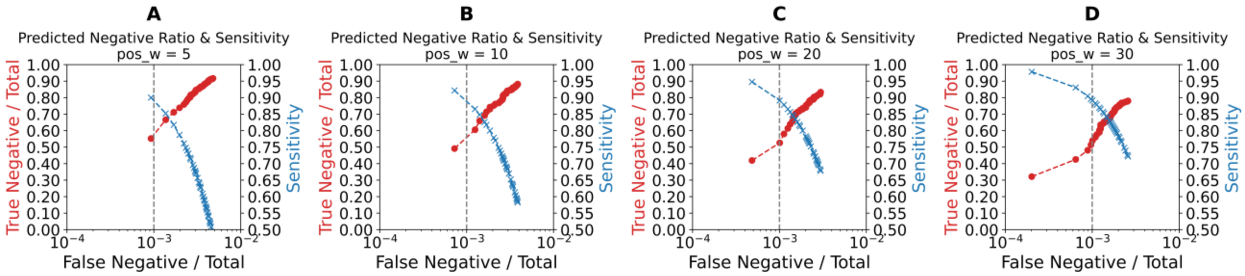

Supplementary Figure 2: Trade-off plots for the ROS1 holdout sets using different positive weights in the loss function: 5 (A), 10 (B), 20 (C), and 30 (D). The x-axis represents the false negative rate (false negatives divided by the total number of cases), while the y-axis represents the true negative rate (true negatives divided by the total cases) for the red plot, and PPA for the blue plot. Increasing the positive weight shifts the blue plot toward the top-left (indicating improved PPA and reduced false negatives) and the red plot toward the bottom-left (indicating reduced false negatives at the expense of lower true negatives).

ALK fusion model performance on the hold out set

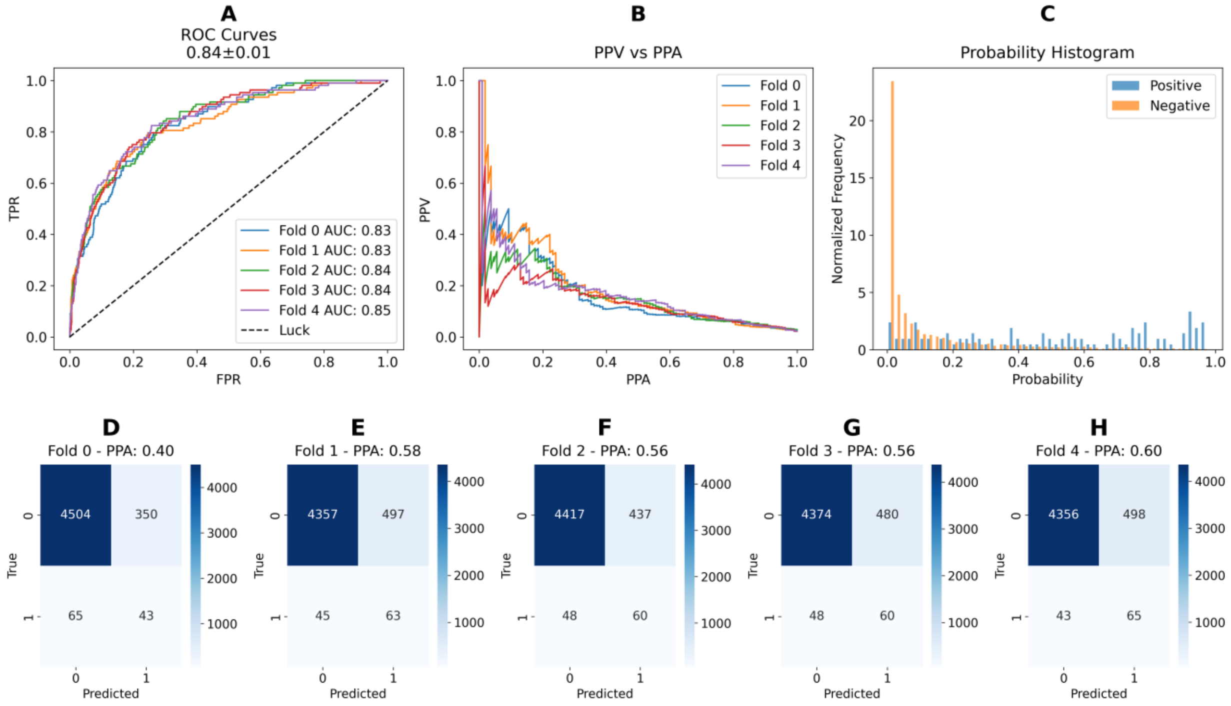

Supplementary Figure 3: Performance plots for ALK on the holdout sets using the train-finetune model. (A) displays the ROC curve, including ROC AUC values for each fold in the legend and the average ± standard deviation ROC AUC at the

top of the plot. (B) shows the precision-recall curves, while (C) illustrates a normalized histogram of predicted probabilities. Panels (D–H) present confusion matrices for each fold, comparing true versus predicted labels and displaying the true positive rate (PPA) at the top of each plot.

Supplementary Table 4: Comparison of the performance of the 5 trained ALK fusion models on test and holdout sets.

	**Fold 0**		**Fold 1**		**Fold 2**		**Fold 3**		**Fold 4**		**Average**	
	Test	***Holdout***	***Test***	***Holdout***	***Test***	***Holdout***	***Test***	***Holdout***	***Test***	***Holdout***	***Test***	***Holdout***
**AUC**	0.85	0.83	0.88	0.83	0.86	0.84	0.81	0.84	0.85	0.85	0.85	0.84
**Accuracy**	0.92	0.92	0.9	0.89	0.9	0.9	0.9	0.89	0.9	0.89	0.90	0.90
**PPA**	0.52	0.4	0.63	0.58	0.6	0.56	0.54	0.56	0.65	0.6	0.59	0.54
**PPV**	0.13	0.11	0.13	0.11	0.12	0.12	0.11	0.11	0.12	0.12	0.12	0.11
**F1-score**	0.21	0.17	0.21	0.19	0.2	0.2	0.19	0.19	0.21	0.19	0.20	0.19
**NPA**	0.93	0.93	0.91	0.9	0.91	0.91	0.91	0.9	0.9	0.9	0.91	0.91

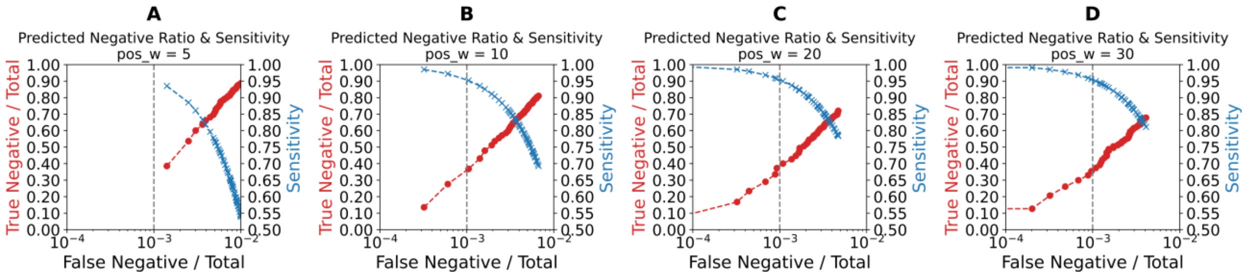

Supplementary Figure 4: Trade-off plots for the ALK holdout sets with varying positive weights in the loss function: 5 (A), 10 (B), 20 (C), and 30 (D). The x-axis shows the false negative rate (false negatives as a proportion of total cases), while the y-axis shows the true negative rate (true negatives as a proportion of total cases) for the red curve and PPA for the blue curve. As the positive weight increases, the blue curve shifts toward the top-left, reflecting enhanced PPA and fewer false negatives, while the red curve moves toward the bottom-left, reflecting a reduction in true negatives
